# Web-Based Visualization of Scientific Research Findings: National-Scale Distribution of Air Pollution in South Korea

**DOI:** 10.3390/ijerph17072230

**Published:** 2020-03-26

**Authors:** Yeonkyeong Park, Insang Song, Jeeeun Yi, Seon-Ju Yi, Sun-Young Kim

**Affiliations:** 1 Department of City and Regional Planning, Cornell University, Ithaca, NY 14850, USA; yp394@cornell.edu; 2 Department of Geography, University of Oregon, Eugene, OR 97403, USA; sigmafelix@hotmail.com; 3 Department of Public Health Sciences, Graduate School of Public Health, Seoul National University, Seoul 08826, Korea; yijee88@snu.ac.kr; 4 Seoul Center for Infectious Diseases Control and Prevention, Seoul Medical Center, Seoul 02053, Korea; yiseonju@snu.ac.kr; 5 Department of Cancer Control and Population Health, Graduate School of Cancer Science and Policy, National Cancer Center, Goyang-si, Gyeonggi-do 10408, Korea

**Keywords:** air pollution, nitrogen dioxide, particulate matter, scientific findings, web-based visualization

## Abstract

Background: As scientific findings of air pollution and subsequent health effects have been accumulating, public interest has also been growing. Accordingly, web visualization is suggested as an effective tool to facilitate public understanding in scientific evidence and to promote communication between the public and academia. We aimed to introduce an example of easy and effective web-based visualization of research findings, relying on predicted concentrations of particulate matter ≤ 10 µg/m^3^ (PM_10_) and nitrogen dioxide (NO_2_) obtained from our previous study in South Korea and Tableau software. Our visualization focuses on nationwide spatial patterns and temporal trends over 14 years, which would not have been accessible without our research results. Methods: Using predicted annual average concentrations of PM_10_ and NO_2_ across approximately 250 districts and maps of administrative divisions in South Korea during 2001–2014, we demonstrate data preprocessing and design procedures in the Tableau dashboard, comprising maps, time-series plots, and bar charts. Results: Our visualization allows one to identify high concentration areas, a long-term temporal trend, and the contrast between two pollutants. The application of easy tools for user-interactive options in Tableau suggests possible easy access to the scientific knowledge of non-experts. Conclusion: Our example contributes to future studies that develop the visualization of research findings in further intuitive designs.

## 1. Introduction

Air pollution is identified as one of the major risk factors to the global burden of mortality and morbidity [1]. This indication is based on the accumulating evidence of epidemiological studies worldwide, that reported the association between long-term exposure to air pollution and various health endpoints [2,3]. As every single individual can be affected by air pollution and the adverse health effect of air pollution was consistently found across different countries in different continents with low to high air pollution concentrations, research findings may attract the interest of researchers and decision makers, as well as the general public. To satisfy these interests, visualization can be a useful tool. 

Visualization of research findings can help enhance our understanding and initiate communications that may help public health actions. Information visualization, particularly based on interactive mapping and spatial analysis tools, often helps one to ‘see’ behaviors and characteristics of phenomena that are otherwise unseen, eliciting the discovery of patterns and relationships that were previously unknown [4,5]. This discovery can trigger visual thinking that can inform decision-making. For example, new knowledge obtained by visual examination for the origin of disease incidence or distribution of limited public health resources may affect the adoption of specific public health policies [6]. The map of spatial distribution of air pollution concentrations also helps identify highly polluted areas and possible pollution sources, and initiates policy efforts to improve air quality in those areas. Data visualization also helps the communication between researchers and public audiences, by engaging people with the research data through the visualization’s content and design [7]. Essential understanding is facilitated by using various charts of familiar types to public (e.g., bar, line, and scatter plots) and maps of static or interactive features [7]. 

In particular, web-based visualization brings us extended benefits. Web-based visualization enables easier access and simple browsing to users ubiquitously [8]. It also allows users to customize applications and data representations, through dynamic linkages between web-based data and visualization applications [9]. Web interaction and navigation techniques in visualization facilitate users to follow associated hyperlinks and to drill down to underlying data in an intuitive point-and-click paradigm [4]. In addition to data exploration, web-based visualization enabled by grid and cloud computing platforms also prompts effective data sharing [8]. Data consumption has expanded to a broader audience, from researchers and decision-makers, to any knowledge-seekers who want to use data to answer their questions [10]. As data become more publicly available on the web, many online data visualization systems appear to serve the demands for such data sharing and data analysis [11]. Local and national government initiatives, such as the Open Data movement [12], further enhance the availability of data to the public.

As web-based visualization is increasingly popular, various web-based development tools are introduced and used widely. Some studies rely on software such as Java Script and Google Earth that require some technical knowledge and skills [13,14]. In contrast, others employ easy applications such as Tableau and Many Eyes, that allow us to manipulate and analyze data with little knowledge in computer languages [11]. Tableau has been one of the popular tools mainly used in the private sector and has gradually expanded its user base to academia and the public sector. For example, Ohio State University Libraries experimented for data visualization and rapid analytics using Tableau [15]. Moreover, international organizations, such as the United Nations (UN), utilized Tableau as a visual analytics standard across the UN system [16]. 

Web-based visualization of air pollution easily implemented by scientists can be a powerful tool to reduce the gap between expert knowledge and public understanding. Although public understanding and awareness is considered as being highly contributable to improving air quality and preventing adverse health effects, previous studies indicate its gap from the scientific knowledge of experts [17,18]. In a survey of 25,525 people in 27 European countries, 59% responded uninformed about air quality issues in their countries [19]. Furthermore, 56% of respondents thought that air quality had deteriorated in the last 10 years, despite the generally improved air quality. In the UK, people tended not to know the level of air pollution in their residential neighborhoods, even for those living close to pollution sources [20]. Some survey studies in China and South Korea also indicated people’s perception of deteriorating air quality over time, which is opposed to actual decreasing trends of pollution concentrations [21,22]. Behind this trend, it is noteworthy that the information service of web visualization for air pollution has been dominantly provided by governmental agencies. This type of service mainly focuses on informing concurrent levels of air pollution based on measurement data, collected in air quality regulatory monitoring networks. One of the major gaps resulting from such visualization would be temporal and spatial patterns, which can be identified based on the comparison of air pollution level at one time point in an area to other periods and/or areas. It is implied that people’s perceptions are generally formulated by what they see on the display rather than the whole story of scientific findings, creating a knowledge gap.

Scientific evidence of temporal and spatial patterns of air pollution achieved by previous studies can be a good example to maximize the benefits of web-based visualization and to fill in the knowledge gap. One of the major limitations in studies of air pollution and human health is the unavailability of air pollution measurements at people’s homes and works. Air pollution measurements are available only at limited monitoring sites implemented by governments for regulatory purposes or individual research projects. To overcome this limitation, many recent studies have developed air pollution prediction models to estimate air pollution concentrations at any locations without measurements. Likewise, our recent study in South Korea developed air pollution prediction models for particulate matter with an aerodynamic diameter less than or equal to 10 μm (PM_10_) and nitrogen dioxide (NO_2_) [23]. Air pollution concentrations estimated at many unmeasured locations in the country, as outputs of this study, would provide a unique opportunity to investigate national, regional, and temporal patterns of air pollution, identify hotspots, investigate potential emission sources, and explore possible policy solutions, when effectively visualized and shared with other researchers and the general public. 

Based on our recent scientific achievement of the air pollution prediction model, this paper aims to (1) present new results using web-based visualization in Tableau, (2) fill the knowledge gap between experts and the public, and (3) provide practical guidance to future research. We specifically intend to explore nationwide spatial patterns and temporal trends over 14 years in South Korea, which have not been accessible relying on existing regulatory monitoring network data alone. We focus on PM_10_ and NO_2_ to maximize the benefit of our visualization. These two pollutants are most commonly applied to prediction models based on their adverse health effects found in many epidemiological and toxicological studies [3,24,25]. Their different characteristics as a regional versus local pollutant can provide interesting contrast in temporal and spatial patterns when visualized. These findings can also help enhance consistent understanding between scientists and the public. Lastly, we demonstrate the detailed procedure of each step from acquisition, processing, and combining of different types of data, design of temporal and spatial patterns in Tableau, and exploration of published patterns. This specific information can guide scientists who want to publish their research results using web visualization on their own.

## 2. Data Acquisition and Processing

### 2.1. Data Type and Acquisition

In order to carry out web-based visualization of PM_10_ and NO_2_ concentrations over South Korea, we used two types of data: annual average concentration of air pollution and a map of administrative division. For air pollution concentration, we used measurement data obtained from the National Institute of Environmental Research [26] and generated prediction data based on our previous study [17]. We included these two types of air pollution data to our visualization, in order to present similarities and differences in their spatial and temporal patterns, resulting from the same original data and different spatial coverage. We obtained hourly measurements at regulatory monitoring network sites and their locations in South Korea for 2001–2014 [27]. This data is also downloadable in AirKorea, the website where real-time air pollution conditions are informed based on air quality regulatory monitoring data (https://www.airkorea.or.kr/web/last_amb_hour_data?pMENU_NO=123). As of the year 2010, there were approximately 300 air quality monitoring sites nationwide in South Korea. However, approximately 40% of the districts do not contain any monitoring sites within the area [28]. Using hourly measurements, we computed annual average concentrations at each site, using the inclusion site criteria that excluded temporally or seasonally running sites [27]. We used the annual average concentration, to focus on the spatial patterns of air pollution concentrations rather than daily or seasonal changes. 

Based on the measurement data, our previous study developed an air pollution prediction model, which allows us to estimate population-representative concentrations of PM_10_ and NO_2_ for 2001–2014. The air pollution prediction model was described elsewhere [23]. This pointwise spatial prediction model was developed with the aim of predicting air pollution concentrations at any location without measurements in South Korea. In brief, the pointwise spatial prediction model of annual average PM_10_ and NO_2_ concentrations was developed in a universal kriging framework, including summary predictors of more than 300 geographic variables and spatial correlation based on the air quality regulatory monitoring data in South Korea. These geographic variables include potential pollution sources such as traffic and land use. The model performance was modest or good (cross-validated R^2^= 0.45 and 0.82 for PM_10_ and NO_2_, respectively) and consistent with other national prediction models of PM_10_ and NO_2_ in the U.S. and Europe [29,30,31]. 

To assess the population-level exposure, we applied this model, predicted the annual average concentration at the centroid of the largest residential area in each of the 83,643 census tracts in South Korea, and aggregated into 245–252 districts (see the Section 2.2.2). We used census tract centroids as prediction points to assess the population-level exposure as shown in the previous studies [32,33,34]. The census tracts are the smallest territorial units, for which the population data are available in South Korea (average population in 2010 = 572; median area = 0.02 km^2^) and many other countries.

**Figure 1:**
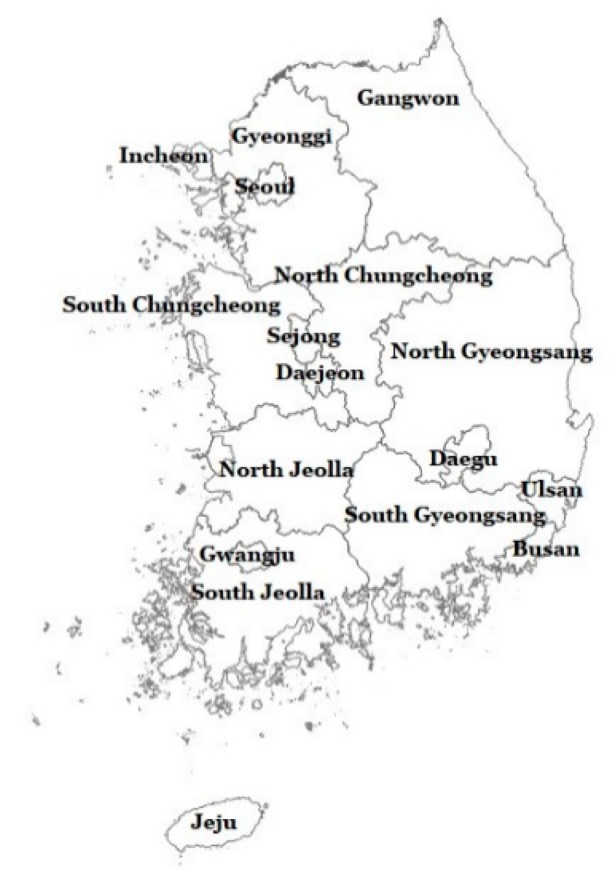
Map of administrative divisions at the provincial level in South Korea (Source: Statistical Geographic Information Service of the Statistics Korea: SGIS).

The maps of the 83,643 census tracts in 2010 and 245–252 districts for 2001–2014 were obtained from the Statistical Geographic Information Service (SGIS) of Statistics Korea (https://sgis.kostat.go.kr/jsp/english/thematic.jsp), as shapefiles in the Geographic Information System. District areas were used to present spatial distribution of air pollution across the corresponding areas each year. The administrative division system of South Korea is comprised of 17 metropolitan cities and provinces at the provincial level (average population in 2014 = 3,008,321, median size in 2014 = 5900 km^2^) and approximately 250 districts at the municipal level (225,293, 442 km^2^) (Table S1 and Figure S1). The provincial-level divisions include eight metropolitan cities, including Seoul, the capital of South Korea, and nine provinces (Figure 1). 

While Tableau provides the default boundary map data from the OpenStreetMap, a crowdsourced web map, this background map is only based on the concurrent year and does not include frequent and complicated changes which occurred over the 14 years between 2001 and 2014. The total number of districts was 245 in the year 2001, which increased to 252 in the year 2014 [35]. A major reason for the changing number of districts are the consolidation or separation of provinces and districts; these provinces and districts were merged into one unified jurisdiction or divided into multiple jurisdictions. For example, Sejong Special Autonomous City, one of the eight metropolitan cities, was established in the year 2012 as a result of the consolidation of three districts of two different provinces, in parts, or as a whole. Thus, we relied on the annual map data in shapefiles obtained from SGIS, to overlay the air pollution data of each year.

### 2.2. Data Processing

Our visualization is based on the annual average concentrations for approximately 250 districts in South Korea. In order to display the air pollution data in accordance to respective geographic boundaries, we made some editions for the map data of geographic information and the tabular data of air pollution. Data were processed in the following steps: editing the map and air pollution data, and then merging the two data. 

#### 2.2.1. Editing the Map Data

We compiled the maps of South Korean provinces and districts over the 14-year period. First, we converted the projected coordinate systems of each year’s data to the identical system using the 2000 Korea Unified Coordinate System, because the original map data were constructed based on different projected coordinate systems each year. After the alignment, we revised the administrative code and official name of each district to ensure consistency across years. District codes and names varied by year, reflecting adjustments in the administrative boundaries by expansions, annexations, and partitions of district areas. In addition, district names particularly showed considerable variations resulting from typos, different numbers of blank spaces between words, or missing a part of names. Finally, 14 different shapefiles were combined in ArcGIS (version 10.4.1; Environmental Systems Research Institute, Inc., Redlands, CA, USA).

#### 2.2.2. Editing the Air Pollution Data

Using measured annual average concentrations of PM_10_ and NO_2_ at air quality regulatory monitoring sites and predicted annual average concentrations at the residential census tract centroids, we computed district averages of annual average concentrations. To compute district averages for measurement data, we were restricted to the regulatory monitoring data collected at urban background monitoring sites and excluded the other three types of sites, to focus on the population level of air pollution. The urban background monitoring sites are located mostly in the municipal buildings in the highly populated areas, with no major pollution sources in the adjacent areas [26]. The other three types of sites, including urban roadside, regional background, and national background sites, are deployed for monitoring air pollution concentrations resulting from traffic, regional, and foreign sources, respectively. After loading monitoring sites and residential census tract centroids with district maps for each year in ArcGIS, we identified the district where each monitoring site and census tract centroid is located. Then, we computed district-specific averages of measurements and predictions each year by two pollutants. In addition, we presented district averages of prediction uncertainties, by using the same approach used for prediction averages. Then, we revised district codes and names to have consistent styles across years, and added English-translated names of provinces and districts for international readers. 

#### 2.2.3. Merging the Two Data

Using the shapefiles of district maps and tabular files of district-specific annual average concentrations for PM_10_ and NO_2_, we combined the two types of data by using the district code of each year. We did not use the district name, because of its large variations in different characters or styles depending on the year and data source. 

## 3. Designing Visualization in Tableau

**Figure 2:**
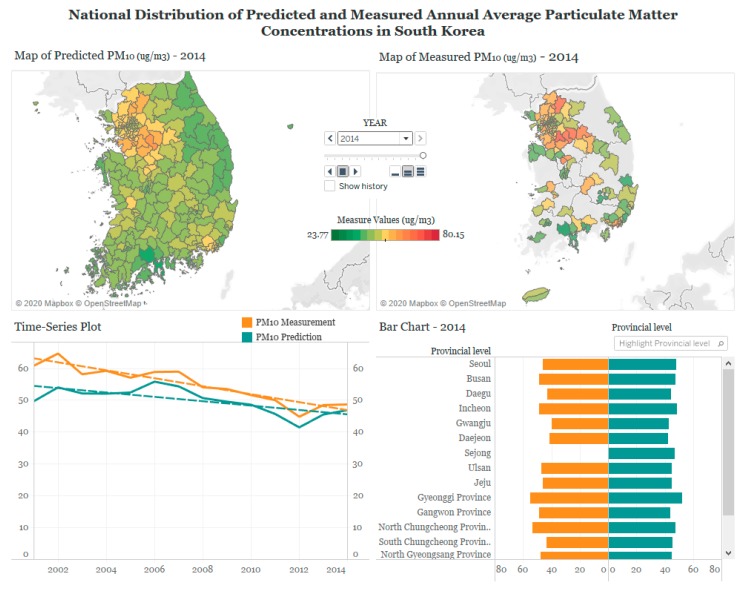
An example of the Tableau dashboard for visualizing measured and predicted annual average particulate matter with an aerodynamic diameter ≤ 10 μm (PM_10_) concentrations (µg/m^3^) in South Korea, 2001–2014 (maps presented above show the concentrations for the year 2014 selected on the ‘pages shelf’ and can be replaced with concentrations in any specific year when selected).

**Figure 3:**
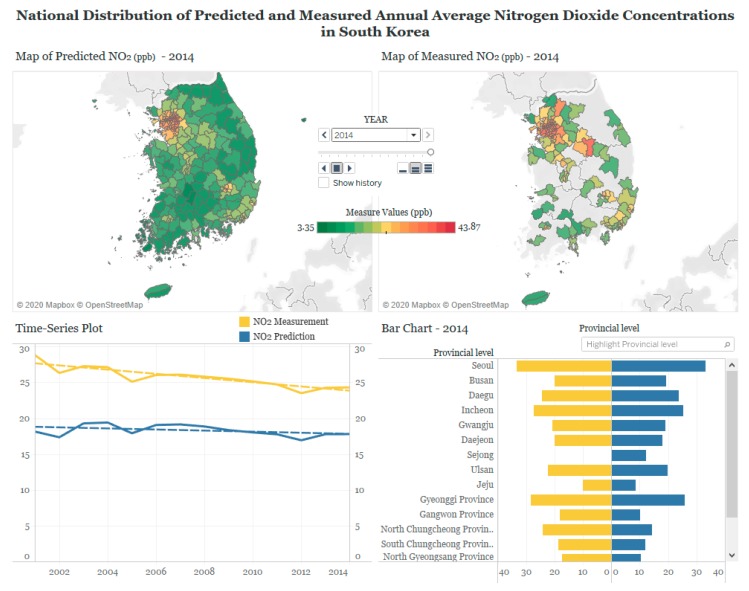
An example of the Tableau dashboard for visualizing measured and predicted annual average nitrogen dioxide (NO_2_) concentrations in South Korea, 2001–2014 (maps presented above show the concentrations for the year 2014 selected on the ‘pages shelf’ and can be replaced with concentrations in any specific year when selected).

As an example of effective visualization of research products, our goal is to visualize the distribution of air pollution to facilitate the understanding in scholastic data and promoting communication between the public and academia. Using the combined set of air pollution and map data, we designed various plots in Tableau to allow users to explore the national, regional, and temporal trends of PM_10_ and NO_2_ in South Korea. These three types of trends were assessed by map, time-series plot, and bar chart, respectively (Table 1). We created three sets of plots and provided different selection tools that allow users to display subsets of the data upon their preference in each plot. Then we arranged the plots on a Tableau dashboard by each pollutant, as shown in Figure 2 and Figure 3. The Tableau dashboard is a collection of several views, such as tables, charts, plots, and maps to compare a variety of data simultaneously [16], displaying user-interactive visual analytics at a glance without any technical computer skills (https://www.tableau.com/learn/training). 

Taking an example of our case using maps, time-series plots, and bar charts (Figure 2 and Table 1), browsing options for readers can be likewise summarized in Figure S2 and Table S2. The map displays the spatial distribution of air pollutant concentrations across all districts of each year, to represent the national trend. The time-series plot demonstrates the temporal trend of air pollutant concentrations over 14 years. The bar chart shows concentrations across 17 metropolitan cities and provinces to inform the regional trend. Such information can be explored using various functions on the dashboards. User-interactive functions show selective information within the data: ‘pages shelf’ for year selection (Figure S2-A), ‘highlight’ for province selection (Figure S3), zoom-in and -out for various spatial scales of visualization, and pop-up windows activated by mouse cursors for detailed information (Figure S4). On the dashboards, all three plots of map, time-series plot, and bar chart are inter-connected. When users click a specific year in the ‘pages shelf’ of the map and a specific province in the ‘highlight’ filter of the bar chart, the selection will be applied to other plots. Detailed explanations on the functions of each map, plot, and chart are further explained below.

### 3.1. Map

The map of 250 districts filled with corresponding annual average concentrations of PM_10_ or NO_2_ illustrates the spatial distribution of air pollution concentrations over the country on a given year. The map helps compare the concentrations across districts and identify the districts with high or low concentrations. Spatial patterns and high concentration areas can also be compared over the years. While illustration of the map for the measured concentrations is restricted to only 60% of the districts that include at least one monitoring site, the map of predicted concentrations across all districts displays the nationwide distribution. As the color scale bar demonstrates the measured and predicted concentrations in the same scale (Figure S2-B), users can compare the two sets of concentrations side by side. Users can zoom-in the map to selectively focus on particular districts as well as zoom-out to see the provincial distribution of air pollution (Figures S2-C and S4). Finally, when users click a specific year in the ‘pages shelf’, the bar chart also automatically displays the pollutant concentrations of the chosen year (Figure S2-A). For predicted concentrations, we also created the maps of prediction uncertainty to present the extent of uncertainty in our modelling results across districts, in addition to the predictions.

### 3.2. Time-Series Plot

The time-series plot demonstrates the overall long-term temporal trend over 14 years, using pollutant concentrations averaged for the entire 250 districts of South Korea each year. The temporal trends of measurements and predictions are overlaid along with best-fitted regression lines that indicate linear trends (Figure S2-E and F). From the pop-up windows of the times-series or regression lines pointed by cursor arrows (Figure S5), users can understand the detailed information such as specific concentrations for each year and goodness-of-fit statistics for the regression lines. 

### 3.3. Bar Chart 

The bar chart illustrates the regional trend at the provincial level, based on annual average concentrations of PM_10_ or NO_2_, averaged to each of the 17 metropolitan cities and provinces on a given year. Upon mouse clicks or cursor arrow pointing, the selected province shows the specific average concentration accordingly in the pop-up window (Figure S6). The highlighter filter enables the dashboard to display the measured and predicted concentrations of the selected province in the maps as well. For example, when the highlight filter of the chart is chosen for Seoul, the map also automatically highlights the area of Seoul, including all the subordinate districts of Seoul (Figures S2-H and S3). The ‘hierarchy’ option allows an instant view on the bar plot that shows concentrations in the districts for the selected province (Figures S2-G and S7).

## 4. Web Publishing in Tableau 

Our Tableau dashboards of PM_10_ and NO_2_ concentrations in South Korea can be accessed via https://tabsoft.co/2T7v6ti, as well as the Quick Response code (Figure S8). While users can explore a variety of visualization methods, our basic purpose is to display the different spatial and temporal patterns of PM_10_ and NO_2_. The dashboards also allow easy comparison between measured and predicted concentrations and between PM_10_ and NO_2_, and an exploration of their similarities and differences (Figure 2 and Figure 3). The map, particularly based on predicted concentrations over the country, demonstrates different spatial patterns by two pollutants, in addition to consistently high concentrations in metropolitan cities for both pollutants. In contrast, the map based on measured concentrations is limited to the districts with at least a monitoring site and makes it difficult to investigate the nationwide distribution. The time-series plot displays a decreasing temporal trend of PM_10_ and NO_2_, based on both measurements and predictions. The bar chart demonstrates patterns of higher annual average concentrations of predicted PM_10_ and NO_2_ in metropolitan cities than those in provinces. 

### 4.1. PM_10_

From the map, PM_10_ displays the broad-scale spatial variability with high concentrations in the western part of South Korea, including the Gyeonggi province and two metropolitan cities of Seoul and Incheon, in addition to the other metropolitan cities. Prediction uncertainty was higher in provinces where the number of monitoring sites are smaller compared to the metropolitan cities. Maps over 14 years show that PM_10_ concentrations decrease in most districts over time, although high concentrations in Seoul and Gyeonggi province are consistent (Figures S9 and S10). The time-series plot also shows the decreasing trend over the years in both measured and predicted concentrations of PM_10_. For the two types of concentrations, measured concentrations are consistently higher than the predicted concentrations in all years. This pattern is possibly because measurement data are obtained from regulatory monitoring sites mostly located in the urban areas. The district-average concentrations calculated based on the measurements at those monitoring sites are likely to be higher than prediction-based averages, computed in the urban as well as rural areas of the entire country. The gap between the measured and predicted concentrations is wider in the early years and became narrower in the recent years. The urban versus rural contrast is more obvious in the bar chart that shows higher concentrations in the metropolitan cities than provinces. 

### 4.2. NO_2_

The map of NO_2_ also shows high concentrations in the metropolitan area of the Seoul and Gyeonggi province and other metropolitan cities, as shown in the map of PM_10_. However, the variation is more local compared to the regional variation for PM_10_. Unlike PM_10_, the spatial distribution and magnitude of NO_2_ concentrations hardly change over the years. This contrast is aligned with previous scientific knowledge of two pollutants. While PM_10_ is considered a regional pollutant affected by emissions of local sources, as well as the atmospheric reaction of emissions originating from distant areas, there is a good understanding of NO_2_ as a local pollutant, indicating the significant role of urban and local emission sources such as traffic. However, prediction uncertainty shows a similar pattern to that of PM_10_. NO_2_ predictions are more uncertain in provinces than in the metropolitan cities. The time-series plot shows slightly decreasing trends over the years. The bar chart displays generally high concentrations in metropolitan cities.

## 5. Discussion

In summary, we demonstrated the nationwide spatial and temporal distributions of two major air pollutants, PM_10_ and NO_2_, over the 14 years, from 2001 through 2014, in South Korea. Using Tableau software, we designed two web-based dashboards of maps, time-series plots, and bar charts by two pollutants. Taking an advantage of research products of our previous study, accompanied by publicly available data, our web-based visualization provides an opportunity to share our scientific findings: the contrast of air pollution between urban and rural areas, decreasing pollution concentrations over the previous 14 years, and similarities and differences between PM_10_ and NO_2_ in their spatial and temporal distributions over the country. These findings would have not been shared if we had not used our research results for web-based visualization. The published results on Tableau display the scientific reality discrepant from, or lacking in, public understanding indicated in the previous literature. Our work also provides a practical guidance for future research that aims for the web publication of their research findings.

Our study is intended to illustrate an example of the web-based visualization of modelled air quality data in a user-interactive design, to share academic achievement with extended target readers beyond academia, including the general public and public authorities. Although enormous scientific findings are accumulating, the understanding and application of research findings have been difficult for non-experts. Technical features of scientific findings were indicated as one of the dominant barriers generating the knowledge gap between experts and the public [18]. As the internet is one of the main sources for air quality awareness by the public, new communication platforms, such as web-based visualization created by scientists can be a new powerful tool [36,37]. With an easy access and handling enabled by the online platform, our Tableau dashboards of maps, time-series plots, and bar charts could help users explore spatial and temporal dimensions of scholarly information on the air pollution of South Korea. Such familiar designs of visualization encourage better understanding of research findings among the public. As a result, sharing scientific results could promote public discourse, by increasing public awareness and interest, which indirectly leads to urging transparent decision making in public policy [38,39]. For instance, in 2016, the Ministry of Transportation of Germany undertook an investigation on the manipulation of emission tests of diesel engines by Daimler, recognizing the criticism by U.S. consumers and the U.S. Environmental Protection Agency (EPA) [40]. Media and public interest in the environmental policy of the U.S. have been developed by U.S. EPA’s reports with scholarly data visualization [41]. Moreover, ‘easy’ data comprehension led by simple manipulation of various visualization tools is certainly critical in academia. It can expedite the sheer speed of interpretation, as well as understanding of complex theories and research outcomes [42]. Such a high quality of interpretation helps scholars self-check their findings, and furthermore, prepare for the healthy proliferation of future studies. 

Our choice of an easy application tool for web-based visualization, without relying on advanced technical skills, promotes the active sharing of research outputs. Among various tools that were developed, we used Tableau, as one example, that was designed to recommend efficient designs of plots, charts, and color schemes for users to understand the given data. It is also user friendly, in that it provides various kinds of intuitive functions for data handling and data storage, without requiring any programming codes and/or cloud setting. It has mostly been adopted in the private sectors, but other examples of Tableau usage are found throughout the public and government sectors. For example, big data analysis by the U.S. Census Engagement Navigator allows users to quickly understand which part of the country had high or low return rates in the 2010 census, and the current demographic makeup of these neighborhoods. The U.S. Air Force Installation and Mission Support Center has also designed Tableau dashboards to better understand the cost of mission infrastructure. The Gyeonggi Infectious Disease Control Center of South Korea is a health agency that has effectively used Tableau dashboards to demonstrate the research findings on various infectious diseases and share the information with the public. 

While other application tools certainly remain as options for future studies, it becomes clearer to observe our focus on the easy communication of scientific findings, when we compare it to other visualization studies. Table S3 shows five recently published studies that focused on the web visualization of scholarly data [13,14,43,44,45]. Most studies were based on the advanced visualization skills, such as JavaScript. Although their visualization includes many more details based on the effective designs, it would be difficult for researchers to adopt those skills to publish their results online on their own. In addition, some studies were limited to demonstrating publicly available data rather than the scientific findings, as opposed to our work, which specifically focuses on sharing the scientific results. 

Our example of web-based visualization also contributes to providing practical guidance to future studies. We demonstrated our data processing procedure prior to web visualization using map and tabular data, which are generated by governments and publicly available. As these types of administrative data are commonly used for research purpose, our work shares our experience to help future studies. For example, administrative boundaries and names change over time and these changes need to be accounted for when temporal changes are visualized, as shown in our example. Our approach applied to air pollution can also be utilized for presenting temporal and spatial patterns of health effect estimates of air pollution, when related research products are produced.

There are other visualization efforts for sharing the scientific findings with the public, mostly employed by government organizations. For air pollution, there are currently visualization websites available, such as AirNow in the U.S. and AirKorea in South Korea. Those visualization sites aim to inform the concentration level at a given time based on the real-time data measured at regulatory monitoring sites. In contrast, our study attempts to provide nationwide spatial and temporal patterns of air quality using our research outputs of predicted concentrations from our previously developed air pollution prediction model, in addition to the measured concentrations. Monitored data alone are not adequate for assessing nationwide patterns of air pollution, as 40% districts of South Korea do not have at least one monitoring site. The benefit of visualization would be even greater when we consider higher air pollution concentrations in South Korea (average concentration of PM_10_ in 2010 based regulatory on monitoring data = 52 μg/m^3^) [23], compared to those in North America and European regions (21–42 μg/m^3^ in 2008–2010) (https://www.who.int/airpollution/data/cities-2011/en/). In addition, the understanding of higher or lower air pollution concentrations in a given area and time compared to the other areas and/or time periods can motivate following public interests in characteristics that derive such changes. Air quality improvement over time could be attributed to specific regulatory actions [46]. Spatial variation may represent different pollution sources or relevant socioeconomic conditions across areas [32,47,48]. Public understanding of the background of air quality changes is particularly important for encouraging public support and/or participation in improving air quality.

Our study includes several limitations, to be addressed for future research. First, because our study focuses on the long-term exposure of air pollution and uses annual average concentrations, we are not able to provide insight into the short-term variation of air pollution. Future studies should examine micro-temporal scale data, focusing on daily and/or seasonal variation. Updated data for extended years after 2014 should also be additionally visualized for exploring longer-term patterns. In addition, functions and sophistication in the data presentation designs are weaker, compared to those developed by experts with a strong background in computer science and web design. However, such simplicity is in fact intentionally devised in our study for the easier manipulation of dashboard functions, regardless of technical backgrounds. Lastly, our web-based visualization focused on the temporal and spatial patterns because there is a knowledge gap among the public and scientists, as indicated in the previous literature. Future studies can undertake extensive interview or surveys based on the specific questionnaires, to identify other differences and to design a web-based publication targeting those topics.

## 6. Conclusions

We recommend a web-based visualization of scholarly data for active research data sharing beyond academia, by using an easy application without advanced technical skills, allowing users’ effective understanding in scientific information. Such approaches will not only lead to the proliferation of related studies, but also improve the public awareness and understanding. 

## Figures and Tables

**Table 1:** Aims, description, and interactive options of three types of plots on the Tableau dashboards for visualizing measured and predicted concentrations of particulate matter with an aerodynamic diameter ≤ 10 μm (PM_10_) and nitrogen dioxide (NO_2_) in South Korea, 2001–2014.

Visualization	Map	Time-Series Plot	Bar Chart
**Aim**	National trend (spatial distribution)	Temporal trend	Regional inter-comparison
**Description**	Annual average concentrations across about 250 districts in each year	National-scale annual average concentrations over 14 years	Annual average concentrations across 17 metropolitan cities and provinces in each year
**Interactive options for users**	• Pages shelf and drop-down list for year selection • Color scale bar to indicate the pollutant concentration values • Tool bar option (zoom in-out, etc.) • Pop-up window to indicate a specific province and district of a specific year	• Color legend to distinguish the pollutants • Pop-up window to indicate a specific year and the goodness-of-fit statistics of the best-fitted regression line	• Pages shelf and drop-down for year selection • Hierarchy option to add or subtract the levels of data between provinces and districts • Highlight option to show the data in the province selected by typing keywords or from a drop-down list • Pop-up window to indicate a specific province or district of a year

## Supplementary Materials

The following are available online at https://www.mdpi.com/1660-4601/17/7/2230/s1, Table S1: Summary statistics of area and population in provincial-level administrative divisions of South Korea in 2010, Table S2: Aim and interactive options of three types of plots on the Tableau dashboards for visualizing measured and predicted concentrations of PM_10_ and NO_2_ in South Korea, 2001–2014 (A to I interactive option linked to a specific visualized example in Figure S2), Table S3: Characteristics of recently published studies that demonstrate web visualization for air pollution data, Figure 1: Map of administrative divisions at the provincial and district levels in South Korea (Source: Statistical Geographic Information Service of the Statistics Korea: SGIS), Figure S2: An example of the Tableau dashboard for visualizing measured and predicted annual average NO_2_ concentrations in South Korea, 2001–2014, with highlights of user-interactive functions (A to H; see Table S2 for their detailed explanation) and step-by-step sample instruction, Figure S3: Highlight option of the bar plot to select a specific area using keywords or select from a drop-down list on the Tableau dashboard of NO_2_ concentrations in South Korea, 2001–2014, Figure S4: Zoom-in and pop-up window of the map for showing pollutant concentrations in a specific province and district on the Tableau dashboard of PM_10_ concentrations in South Korea, 2001–2014, Figure S5: Time-series lines, best-fitted regression lines, and pop-up windows of goodness-of-fit statistics and the concentration value for a specific year in the time-series plot on the Tableau dashboard of NO_2_ concentrations in South Korea, 2001–2014, Figure S6: Pop-up window for selecting a specific province in bar chart on the Tableau dashboard of NO_2_ concentrations in South Korea, 2001–2014, Figure S7: Hierarchy option to add or subtract air pollution concentrations between the province and district in the bar chart on the Tableau dashboard of NO_2_ concentrations in South Korea, 2001–2014, Figure S8. Quick Response code for the Tableau dashboard for visualizing measured and predicted annual average concentrations of PM_10_ and NO_2_ in South Korea, 2001–2014, Figure S9: Maps of measured and predicted concentrations of PM_10_ across 250 districts in 2002, 2007, and 2014 on the Tableau dashboard of PM_10_ concentrations in South Korea, 2001–2014, Figure S10: Maps of measured and predicted concentrations of NO_2_ across 250 districts in 2002, 2007, and 2014 on the Tableau dashboard of NO_2_ concentrations in South Korea, 2001–2014.
