# Supplementary material for: Web-Based Visualization of Scientific Research Findings: National-Scale Distribution of Air Pollution in South Korea
Source: Int J Environ Res Public Health. 2020 Mar 26;17(7):2230. doi: 10.3390/ijerph17072230 (PMC7177515; doi:10.3390/ijerph17072230)
Supplement: Supplementary file 1 [file ijerph-17-02230-s001.pdf]

Supplementary Materials

Table S1. Summary statistics of area and population in provincial-level administrative divisions of South Korea in 2010

	**Name**	**N of districts** (Total 251)	**Area size (km²)** Total (100,033.08)	**Population** Total (48,580,293)
8 metropolitan cities	Seoul	25	605.25	9,794,304
	Busan	16	767.35	3,414,950
	Daegu	8	884.10	2,446,418
	Incheon	10	1029.44	2,662,509
	Gwangju	5	501.24	1,475,745
	Daejeon	5	539.90	1,501,859
	Ulsan	5	1058.95	1,082,567
	Sejong[1]	0	464.90	210,884
9 provinces	Gyeonggi	44	10167.15	11,379,459
	Gangwon	18	16693.08	1,471,513
	North Chungcheong	13	7433.19	1,512,157
	South Chungcheong	17	8630.25	2,028,002
	North Jeolla	15	8066.76	1,777,220
	South Jeolla	22	12246.82	1,741,499
	North Gyeongsang	24	19028.02	2,600,032
	South Gyeongsang	22	10532.73	3,160,154
	Jeju	2	1848.85	531,905

[1] All summaries except Sejong City were obtained from the 2010 Census; Sejong City was created in 2012 and summaries were obtained from the 2015 Census.

Table S2. Aims and interactive options of three types of plots on the Tableau dashboards for visualizing measured and predicted concentrations of PM_{10} and NO_2 in South Korea, 2001-2014 (A to I interactive option linked to a specific visualized example in Figure S2)

	**Map**	**Time-series Plot**	**Bar Chart**
**Aim**	National trend (spatial distribution)	Temporal trend	Regional intercomparison
**Description**	Annual average concentrations across about 250 districts in each year	National-scale average concentrations over 14 years	Annual average concentrations across 17 metropolitan cities and provinces in each year
**Interactive options for users**	• Pages shelf and drop-down list for year selection (**A**) [1 2]<br>• Color scale bar to indicate the pollutant concentration values (**B**)<br>• Tool bar option (zoom in-out, etc.) (**C**) [1]<br>• Pop-up window to indicate a specific province and district of a specific year (**D, Figure S3**) [1]	• Color legend to distinguish the pollutants (**E**) [1]<br>• Pop-up window to indicate a specific year and the goodness-of-fit statistics of the best-fitted regression line (**F, Figure S4**) [1]	• Pages shelf and drop-down for the year selection (**A**) [1 2]<br>• Hierarchy option to add or subtract the levels of data between provinces and districts (**G, Figure S6**) [1]<br>• Highlight option to show the data in the province selected by typing keywords or from a drop-down list (**H, Figure S2**) [1 2]<br>• Pop-up window to indicate a specific province or district of a year (**I, Figure S5**) [1]
**Related figures**	Figures S3	Figures S4	Figure S2, S5, S6

[1] Each option indicated in the alphabetical character of red boxes

[2] Filters in sync with the rest of the view of the dashboard

Table S3. Characteristics of recently published studies that demonstrate web visualization for air pollution data

Characteristics		Li et al. (2016)	Liu et al. (2018)	Zhou et al. (2016)	Kim et al. (2016)	Li et al. (2017)
**Study feature**	**Aim**	Visualizing Air Quality Index (AQI)	Visualizing intercity correlation of $PM_{2.5}$ time series	Mapping the spatiotemporal patterns of air quality	Visualizing real-time air quality	Estimating population exposure to $PM_{2.5}$
	**Study area**	Beijing, China	Beijing-Tianjin-Hebei region, China	3 Economic Zones of China	Seoul, South Korea	U.S.
	**Time period**	2009-2014	2014	2014	-	2009
	**Pollutant**	$PM_{2.5}$	$PM_{2.5}$	SO_2, NO_2, CO, O_3, PM_{10}, $PM_{2.5}$	SO_2, NO_2, CO, O_3, PM_{10}, $PM_{2.5}$	$PM_{2.5}$, SO_2, NO_2, CO, O_3, PM_{10}
	**Time scale**	Hourly, daily, monthly	Hourly	Daily	Hourly	Daily
**Visual presentation**	**Method**	Map, scatter plot, calendar view	Line chart, matrix	Map, calendar view,	Flow chart	Scatter plot, line plot
	**Software/ language**	Java script (D3: Data-Driven Documents)	ArcGIS, Python, Matplotlib	Python, ArcGIS, CorelDRAW	KML (Keyhole Markup Language), Google Earth	ArcGIS, Java script, Google Maps API (Application Program Interface)
	**Audience for visualization**	Researchers and public	Researchers and public	Researchers and possibly public	Researchers and public	Researchers and possibly public
	**Strength**	Intuitive visualization method, reliable data quality checking, open source tools with open codes available for free	Visualization of a specific scientific finding (e.g. $PM_{2.5}$ movement in the region)	Identification of major air pollution issues, raising public awareness, policy discussion	3D visualization	Real-time web publication
	**Weakness**	Advanced coding, insufficient scientific background (e.g. need of AQI)	Advanced coding, insufficient information on patterns	Insufficient scientific findings	Complexity of KML coding	Advanced coding, insufficient scientific background

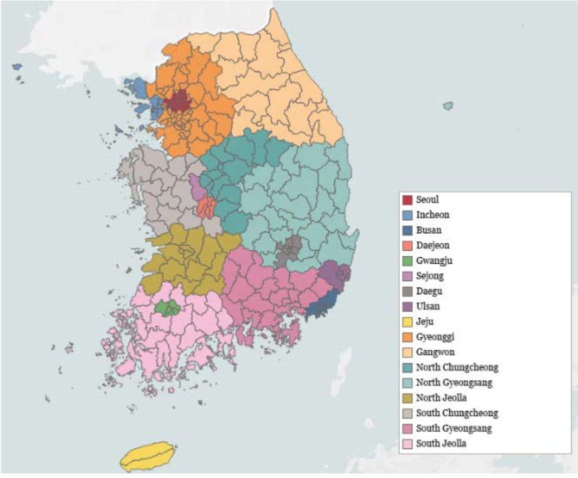

Figure 1. Map of administrative divisions at the provincial and district levels in South Korea (Source: Statistical Geographic Information Service of the Statistics Korea: SGIS).

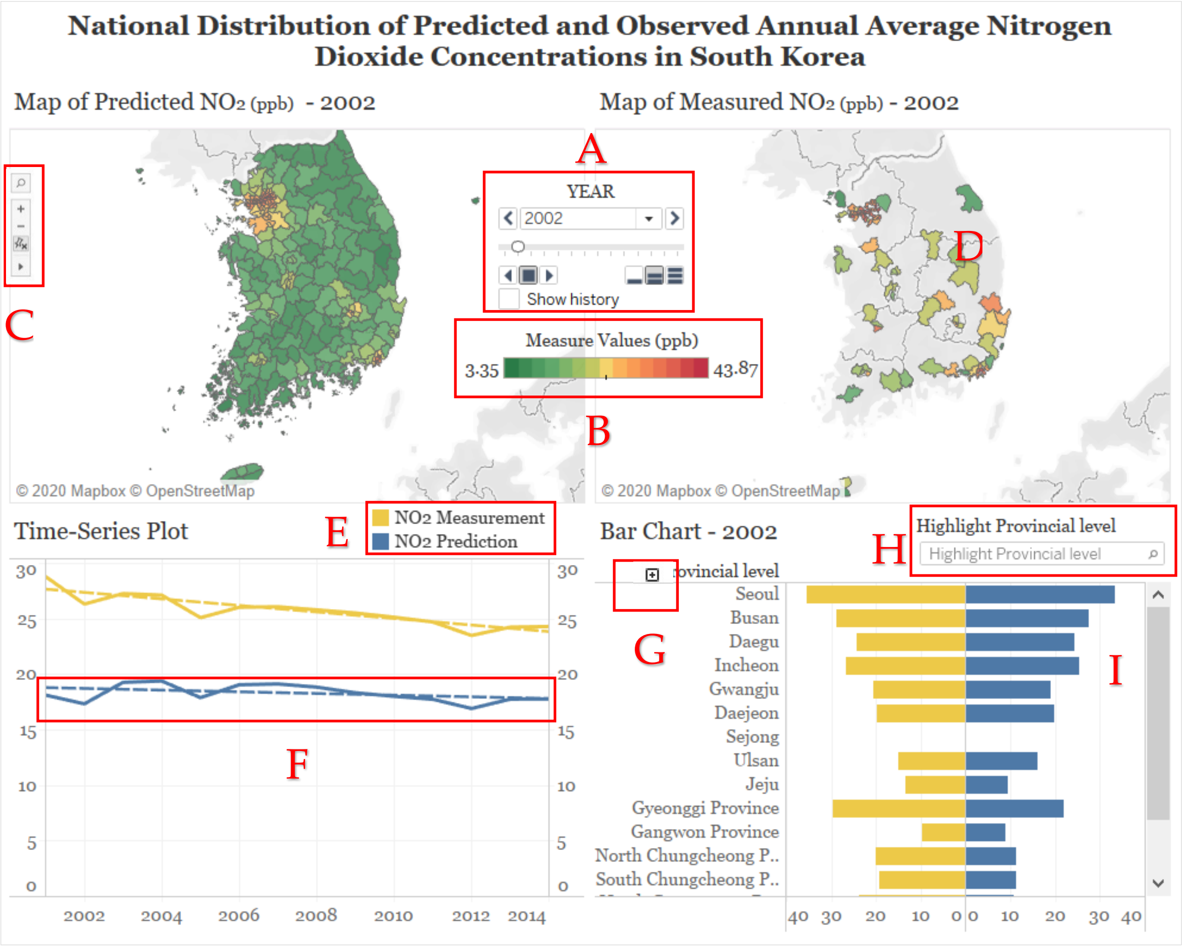

[Step-by-step sample instruction]

To find out the NO_2 concentration in "Jongno-gu" (district), "Seoul" (metropolitan city) in the year 2005:

Step 1. In the 'page shelf' of the map (red box A), click year 2002.

Step 2. Go to the 'highlight' option of the bar chart (red box H), and type "Seoul".

Step 3. Click the 'hierarchy' option, scroll down the list of districts for "Seoul", and find "Jongno-gu".

Step 4. In the concentration bars for NO_2 (I), click the bar for "Jongno-gu".

Figure S2. An example of the Tableau dashboard for visualizing measured and predicted annual average NO_2 concentrations in South Korea, 2001-2014, with highlights of user-interactive functions (A to H; see Table S2 for their detailed explanation) and step-by-step sample instruction

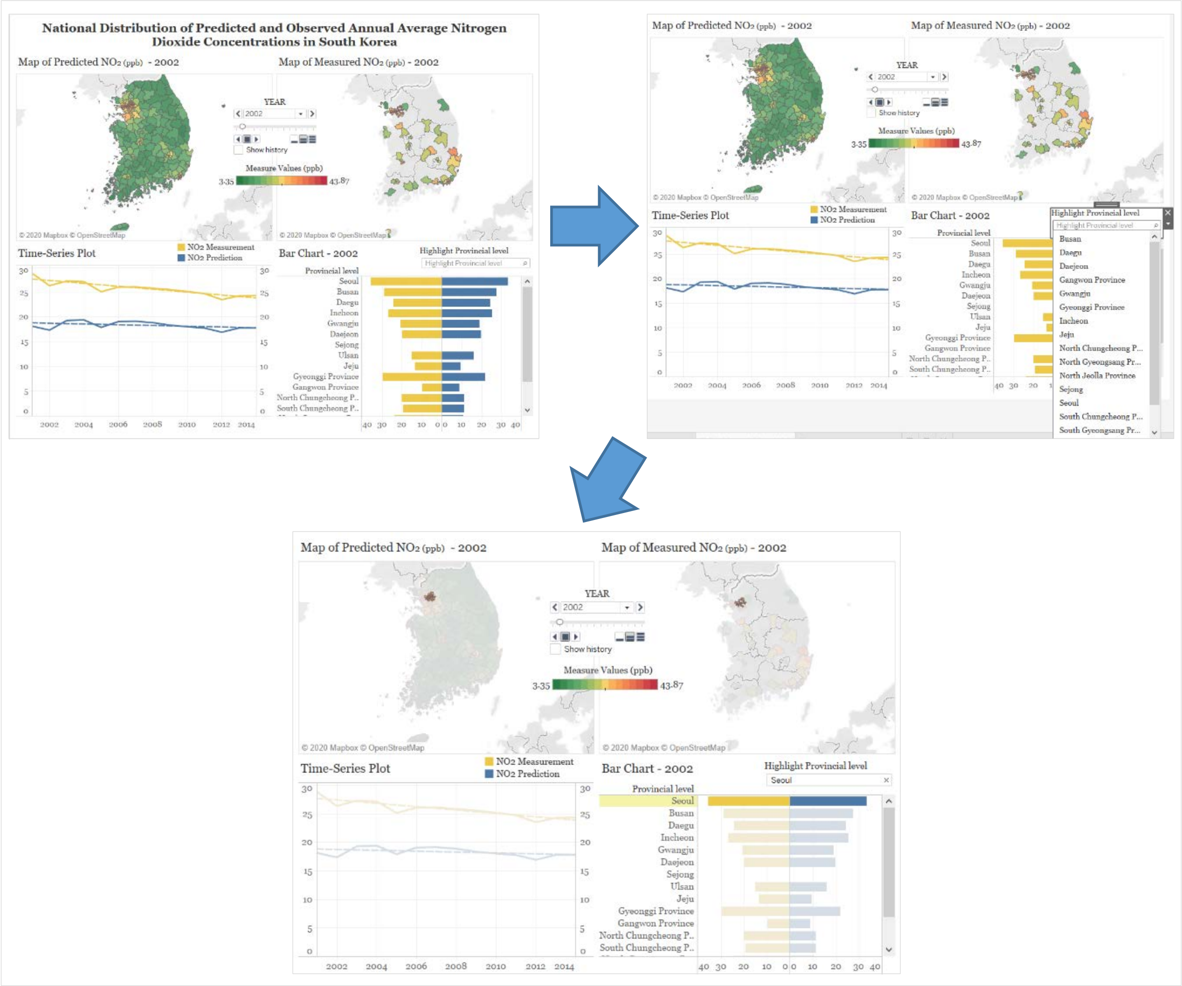

Figure S3. Highlight option of the bar plot to select a specific area using keywords or select from a drop-down list on the Tableau dashboard of NO_2 concentrations in South Korea, 2001-2014

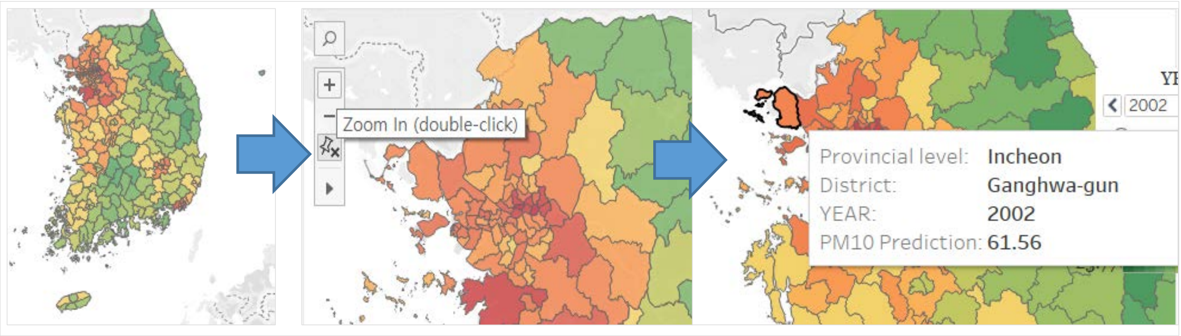

Figure S4. Zoom-in and pop-up window of the map for showing pollutant concentrations in a specific province and district on the Tableau dashboard of PM_{10} concentrations in South Korea, 2001-2014

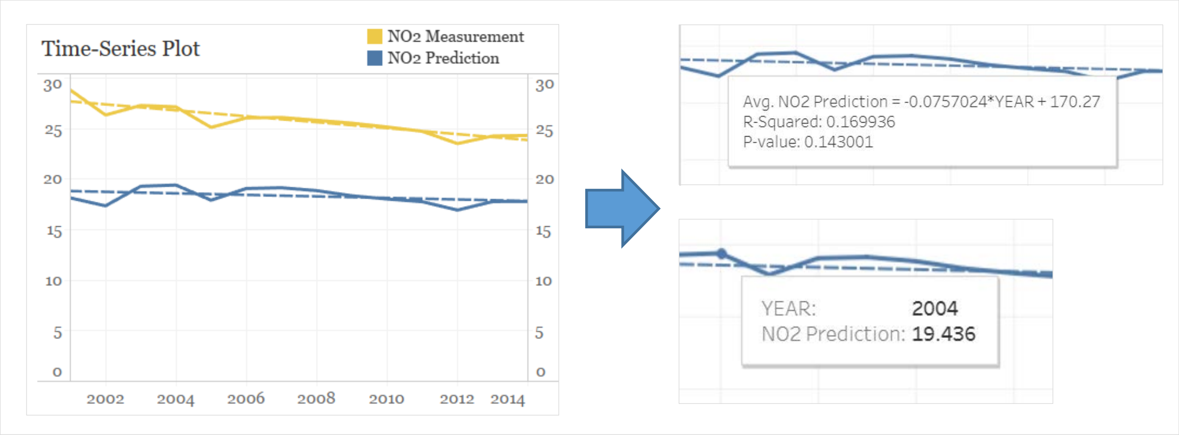

Figure S5. Time-series lines, best-fitted regression lines, and pop-up windows of goodness-of-fit statistics and the concentration value for a specific year in the time-series plot on the Tableau dashboard of NO_2 concentrations in South Korea, 2001-2014

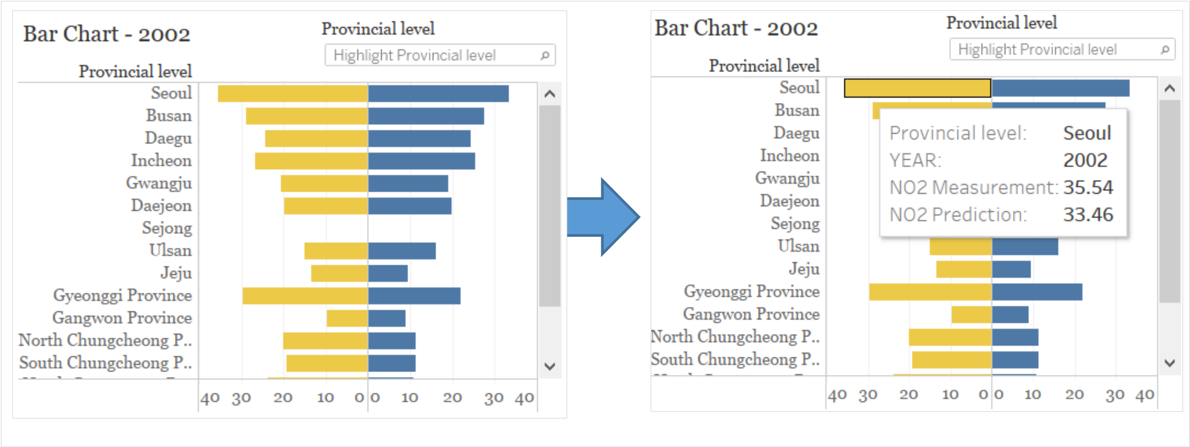

Figure S6. Pop-up window for selecting a specific province in bar chart on the Tableau dashboard of NO_2 concentrations in South Korea, 2001-2014

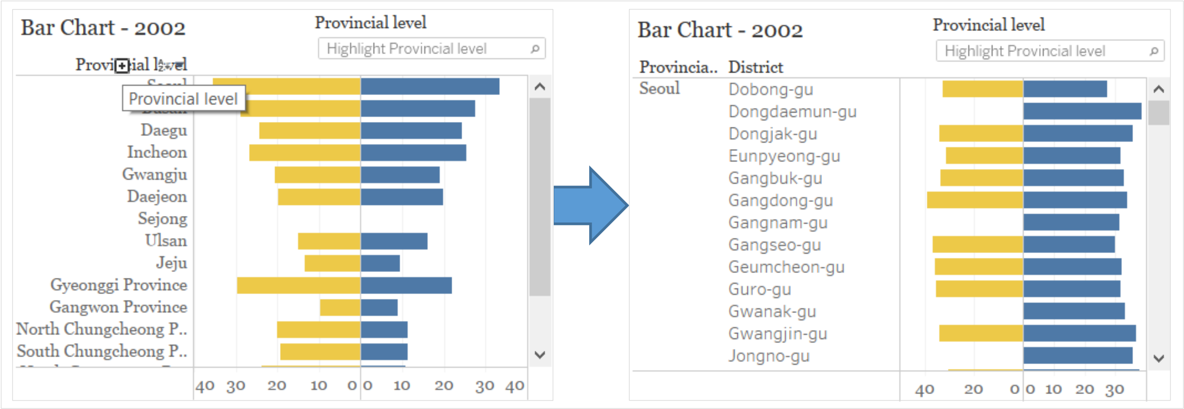

Figure S7. Hierarchy option to add or subtract air pollution concentrations between the province and district in the bar chart on the Tableau dashboard of NO_2 concentrations in South Korea, 2001-2014

Figure S8. Quick Response code for the Tableau dashboard for visualizing measured and predicted annual average concentrations of PM_{10} and NO_2 in South Korea, 2001-2014.

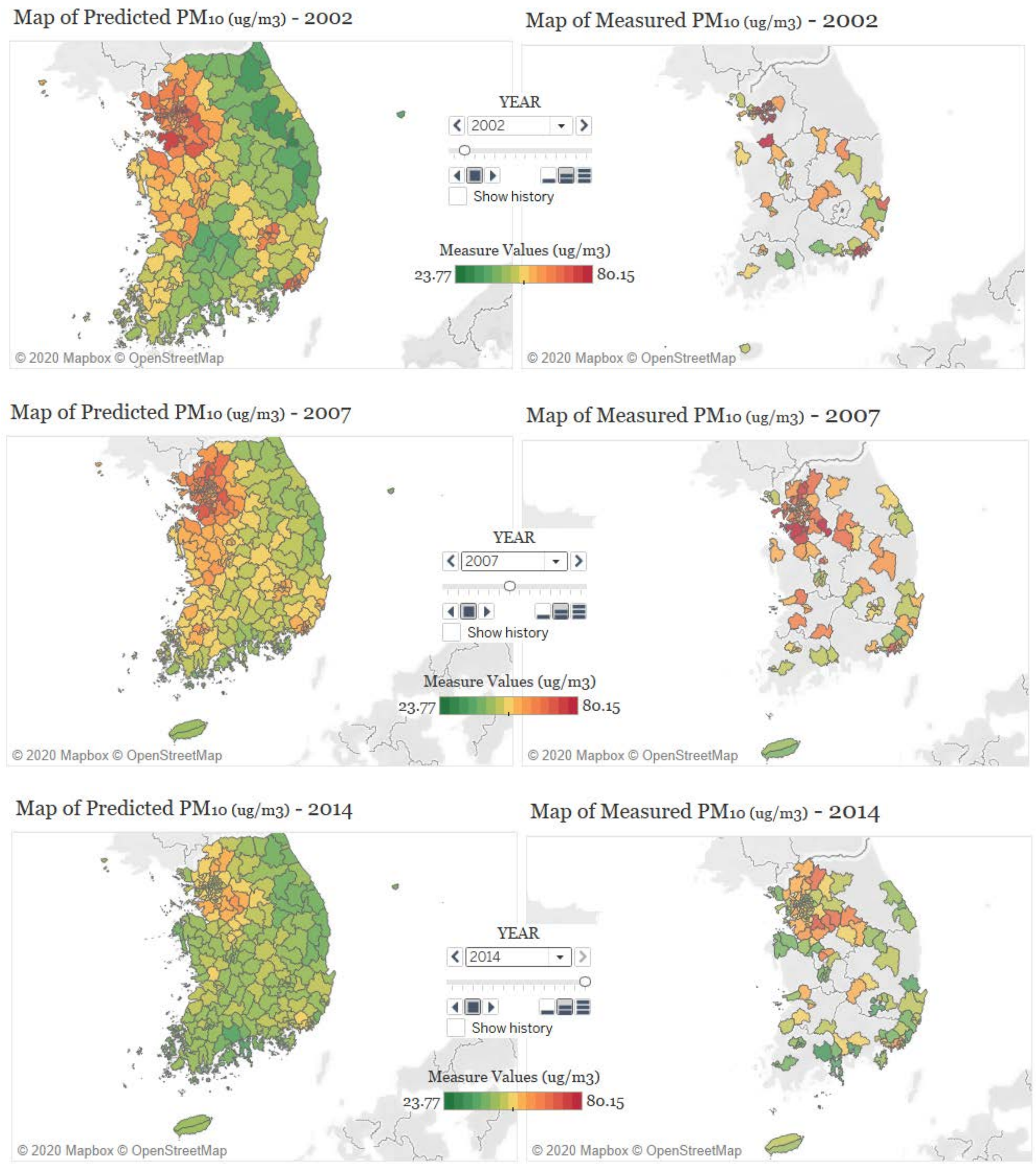

Figure S9. Maps of measured and predicted concentrations of PM_{10} across 250 districts in 2002, 2007, and 2014 on the Tableau dashboard of PM_{10} concentrations in South Korea, 2001-2014

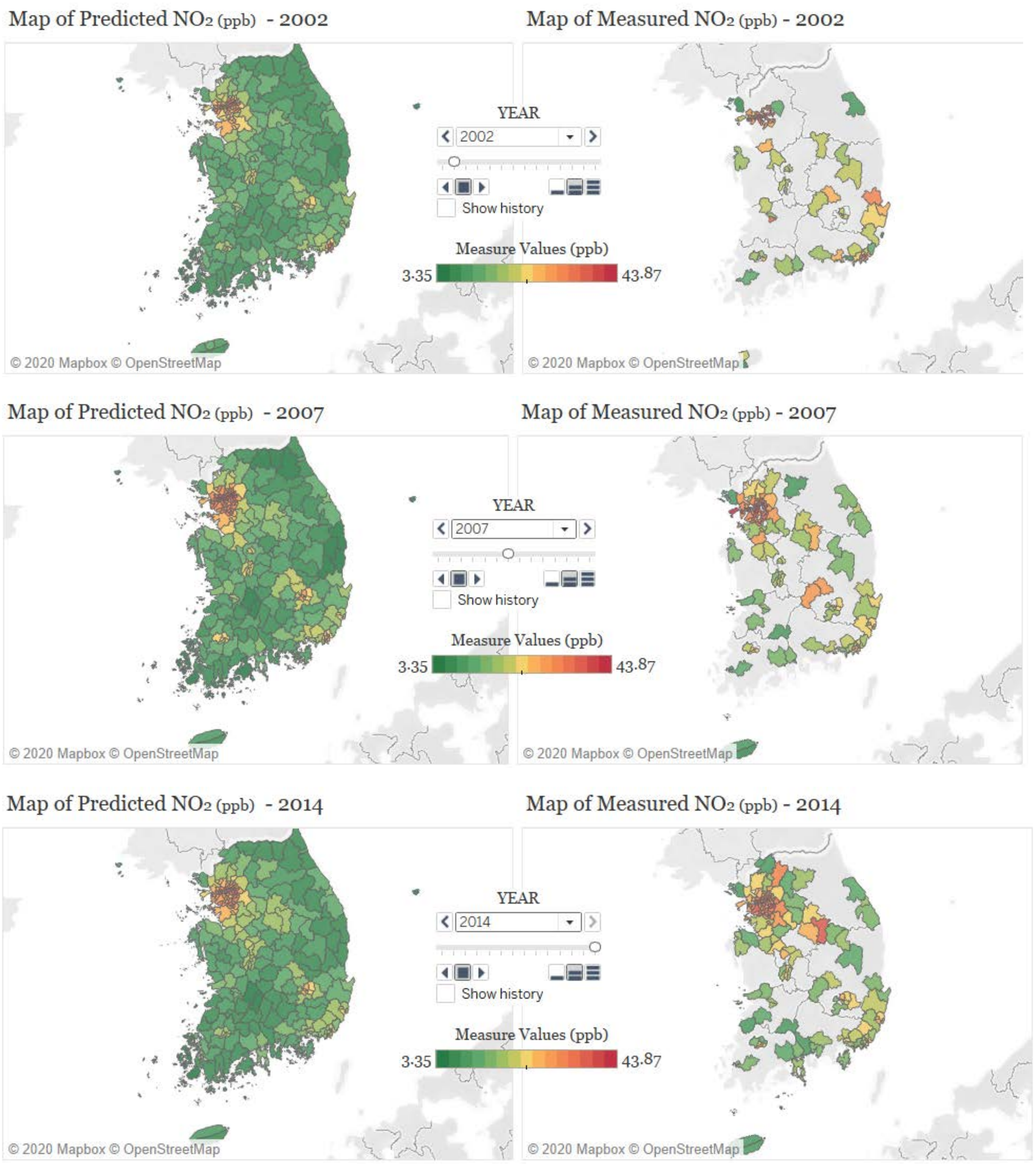

Figure S10. Maps of measured and predicted concentrations of NO_2 across 250 districts in 2002, 2007, and 2014 on the Tableau dashboard of NO_2 concentrations in South Korea, 2001-2014
